# Characterization of the complete mitochondrial genome of *Agapornis personatus* and its phylogenetic analysis

**DOI:** 10.1080/23802359.2019.1681325

**Published:** 2019-10-24

**Authors:** Yun-Xia Chen, Sen-Lin Hou, Yong-Wu Zhou, Ya-Lin Huang

**Affiliations:** a Nanjing Forest Police College, Nanjing, China;; b Key Laboratory of Wildlife Evidence Technology State Forest and Grassland Administration, Nanjing, China

**Keywords:** *Agapornis personatus*, yellow-collared lovebird, mitochondrial genome

## Abstract

In this study, we characterized the complete mitochondrial genome of *Agapornis personatus*. Its mitochondrial genome is a circular molecule of 16,722 bp, with all genes exhibiting typical mitochondrial gene arrangement and transcribing directions. The overall base composition is 21.49% T, 34.6% C, 29.66% A, and 14.25% G. Phylogenetic analysis of the complete mitogenome was conducted using the neighbour-joining method based on 16 parrot species. Phylogenetic tree suggested that *A. personatus* was closest to the same genus specie *Agapornis roseicollis*. The results would be useful for further studies on molecular evolution and breeding works of *A. personatus*.

The parrots of genus *Agapornis* are famous for their affectionate nature, also are called lovebird. Because of their bright and colourful feathers, it is often caught and bred in captivity, resulting in fewer and fewer wild species (Frynta et al. 2010; Li and Jiang 2014; IUCN 2018). *Agapornis personatus* (yellow-collared lovebird) is mainly distributed in the central, northern and eastern coasts of Tanzania, also distributed in the south of Kenya, southwest of Zambia, and northwest of Zimbabwe (IUCN 2018). This species inhabits tropical jungle and grasslands, including the pine forest, riparian forest, acacia forest, and fig forest (Soobramoney and Perrin 2014; IUCN 2018). As they are easy to breed, they have long been human pets and can be found in pet stores around the world. Here, we characterized the complete mitochondrial genome of *A. personatus*.

Captive-bred specimen of *A. personatus* was sampled from the Nanjing Hongshan Forest Zoo (N32°09′, E118°80′), Jiangsu province, China. Whole blood sample was collected from the individual and stored in the Forest Police Forensic Centre of State Forestry Administration (Accession S2019J1101201). DNA extraction, PCR reaction and product purification were carried out by the DNAiso reagent, Ex Taq, and Minibest agarose gel DNA extraction kit, respectively (Takara, Beijing, China). Genome information was obtained through Sanger sequencing.

The complete mitochondrial genome (GenBank accession: MN481404) of *A. personatus* is 16,722 bp in length. The overall base composition of the genome is 21.49% T, 34.6% C, 29.66% A, 14.25% G, and with A + T content of 51.15%. The mitogenome consists of 22 transfer RNA genes, 13 protein-coding genes, 2 ribosomal RNA genes, and one1 control region. The structure and gene arrangement of the mitochondrial genome of *A. personatus* is identical to other lovebirds (Eberhard and Wright 2016; Liu et al. 2019).

To study the phylogenetic relationship of *A. personatus*, the phylogenetic tree was constructed using the neighbour-joining (NJ) methods. Sixteen parrots species for which complete mitochondrial genomes were published are selected from GenBank to assess the genetic and phylogenetic relationship with *A. personatus*. Sequence dataset was aligned using ClustalX and analyzed using the kimura 2-parameter model in MEGA 7.0, with 1000 bootstrap replicates (Kumar et al. 2016). As shown in the phylogenetic NJ tree result (Figure 1), the mitogenome of *A. personatus* was clustered and closest with *Agapornis roseicollis* (EU410486.1). The genome information obtained here could contribute to the genetic diversity conservation of *A. personatus*.

**Figure 1.**
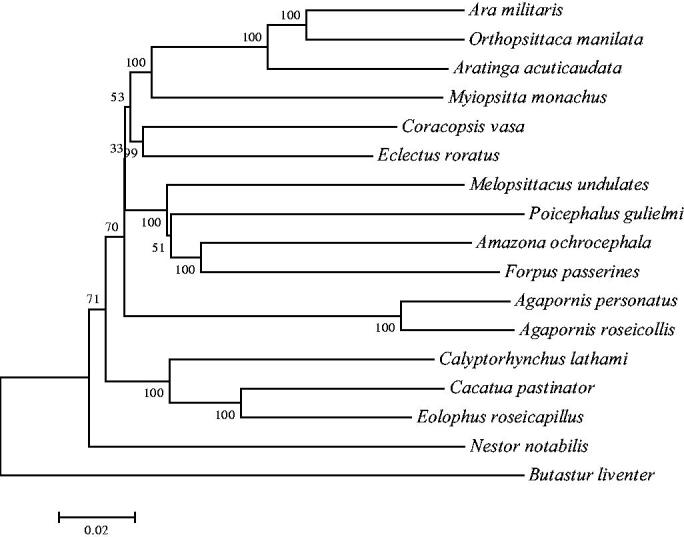
The neighbour-joining (NJ) phylogenetic tree from 16 parrots species mitochondrial genomes with *A. personatus*. Mitochondrial genomes of 16 parrots species have been deposited in the GenBank, the accession numbers are as follows: *Agapornis roseicollis* (EU410486.1), *Amazona ochrocephala* (KM611467.1), *Ara militaris* (KM611466.1), *Aratinga acuticaudata* (JQ782214.1), *Butastur liventer* (AB830617.1), *Cacatua pastinator* (NC_040142.1), *Calyptorhynchus lathami* (JF414241.1), *Coracopsis vasa* (KM611468.1), *Eclectus roratus* (KM611469.1), *Eolophus roseicapillus* (NC_040154.1), *Forpus passerines* (KM611470.1), *Melopsittacus undulates* (EF450826.1), *Myiopsitta monachus* (NC_027844.1), *Nestor notabilis* (MH133967.1), *Orthopsittaca manilata* (KJ579139.1), *Poicephalus gulielmi* (MF977813.1).
